# Modeling Kaempferol as a Potential Pharmacological Agent for COVID-19/PF Co-Occurrence Based on Bioinformatics and System Pharmacological Tools

**DOI:** 10.3389/fphar.2022.865097

**Published:** 2022-06-08

**Authors:** Yong Jiang, Yi-Zi Xie, Chen-Wen Peng, Kai-Nan Yao, Xue-Ying Lin, Shao-Feng Zhan, Hong-Fa Zhuang, Hui-Ting Huang, Xiao-Hong Liu, Xiu-Fang Huang, Hang Li

**Affiliations:** ^1^ Shenzhen Hospital of Integrated Traditional Chinese and Western Medicine, Shenzhen, China; ^2^ The First Affiliated Hospital of Guangzhou University of Chinese Medicine, Guangzhou, China; ^3^ The First Clinical Medical School, Guangzhou University of Chinese Medicine, Guangzhou, China; ^4^ Shenzhen Bao’an District Traditional Chinese Medicine Hospital, Guangzhou University of Chinese Medicine, Shenzhen, China; ^5^ Lingnan Medical Research Center of Guangzhou University of Chinese Medicine, Guangzhou, China

**Keywords:** kaempferol, pulmonary fibrosis, COVID-19, co-occurrence, bioinformatic analysis, system pharmacology

## Abstract

**Objective:** People suffering from coronavirus disease 2019 (COVID-19) are prone to develop pulmonary fibrosis (PF), but there is currently no definitive treatment for COVID-19/PF co-occurrence. Kaempferol with promising antiviral and anti-fibrotic effects is expected to become a potential treatment for COVID-19 and PF comorbidities. Therefore, this study explored the targets and molecular mechanisms of kaempferol against COVID-19/PF co-occurrence by bioinformatics and network pharmacology.

**Methods:** Various open-source databases and Venn Diagram tool were applied to confirm the targets of kaempferol against COVID-19/PF co-occurrence. Protein-protein interaction (PPI), MCODE, key transcription factors, tissue-specific enrichment, molecular docking, Gene ontology (GO) and Kyoto Encyclopedia of Genes and Genomes (KEGG) enrichment analyses were used to clarify the influential molecular mechanisms of kaempferol against COVID-19 and PF comorbidities.

**Results:** 290 targets and 203 transcription factors of kaempferol against COVID-19/PF co-occurrence were captured. Epidermal growth factor receptor (EGFR), proto-oncogene tyrosine-protein kinase SRC (SRC), mitogen-activated protein kinase 3 (MAPK3), mitogen-activated protein kinase 1 (MAPK1), mitogen-activated protein kinase 8 (MAPK8), RAC-alpha serine/threonine-protein kinase (AKT1), transcription factor p65 (RELA) and phosphatidylinositol 4,5-bisphosphate 3-kinase catalytic subunit alpha isoform (PIK3CA) were identified as the most critical targets, and kaempferol showed effective binding activities with the above critical eight targets. Further, anti-COVID-19/PF co-occurrence effects of kaempferol were associated with the regulation of inflammation, oxidative stress, immunity, virus infection, cell growth process and metabolism. EGFR, interleukin 17 (IL-17), tumor necrosis factor (TNF), hypoxia inducible factor 1 (HIF-1), phosphoinositide 3-kinase/AKT serine/threonine kinase (PI3K/AKT) and Toll-like receptor signaling pathways were identified as the key anti-COVID-19/PF co-occurrence pathways.

**Conclusion:** Kaempferol is a candidate treatment for COVID-19/PF co-occurrence. The underlying mechanisms may be related to the regulation of critical targets (EGFR, SRC, MAPK3, MAPK1, MAPK8, AKT1, RELA, PIK3CA and so on) and EGFR, IL-17, TNF, HIF-1, PI3K/AKT and Toll-like receptor signaling pathways. This study contributes to guiding development of new drugs for COVID-19 and PF comorbidities.

## Introduction

The outbreak of coronavirus disease 2019 (COVID-19) in December 2019, with rising incidence and prevalence worldwide, has caused more than six million deaths (World Health Organization, 2022). Severe acute respiratory syndrome coronavirus-2 (SARS-CoV-2) is the trigger for COVID-19 pandemic, and belongs to the same coronavirus lineage that causes SARS (Zhu et al., 2020). Common clinical symptoms of SARS-CoV-2 infection include fever, cough, tiredness, shortness of breath and even death with exacerbation (Wu and McGoogan, 2020). Independent risk factors associated with COVID-19 include hypertension, diabetes, chronic obstructive pulmonary disease, and cardiovascular and cerebrovascular diseases (Wang et al., 2020). Although vaccine use has reduced the incidence of COVID-19, vaccinated people are still at risk of contracting SARS-CoV-2 and the number of COVID-19 cases remains high (Soleimanpour and Yaghoubi, 2021). Drugs against SARS-CoV-2 have been developed that reduce the risk of COVID-19 developing into severe COVID-19, but drug-resistant variants of SARS-CoV-2 may still emerge (Hammond et al., 2022). These shows that COVID-19 remains a serious threat to global health.

Pulmonary fibrosis (PF) is a pathological event caused by acute and chronic interstitial lung injury. PF causes chronic dyspnea, long-term disability and affects the quality of life of the patients (Lechowicz et al., 2020). PF is characterized by alveolar epithelium damage, inflammation infiltration, myofibroblasts activation and excessive deposition of extracellular matrix (ECM) (Giacomelli et al., 2021). Of note, CT images of 62 COVID-19 patients in Wuhan show vacuolar sign in more than half of them (Zhou et al., 2020). Diffuse alveolar damage, fibroblast proliferation and fibrosis are also found in autopsies of COVID-19 patients (Schaller et al., 2020). Alveolar epithelial type II (ATII) cells show a decreasing trend in SARS-CoV-2 infected patients (Delorey et al., 2021). The spike (S) protein of SARS-CoV-2 binds to angiotensin-converting enzyme 2 (ACE2) expressed in ATII cells to enter host cells (Ziegler et al., 2020; Celik et al., 2021). Damaged ATII cells can release transforming growth factor-β (TGF-β) (Tatler and Jenkins, 2012), platelet derived growth factor (Antoniades et al., 1990), connective tissue growth factor (Pan et al., 2001) and interluekin-6 (IL-6) (Crestani et al., 1994), thereby activating lung fibroblasts to increase ECM deposition and promote the development of PF (Sisson et al., 2010). The above researches reveal that COVID-19 patients are at high risk of developing PF (George et al., 2020). Obviously, COVID-19/PF co-occurrence is a catastrophic threat to global health, and it is unclear whether the damage caused by COVID-19/PF co-occurrence can be reversed (John et al., 2021). Therefore, it is an urgent need to find an influential treatment for COVID-19/PF co-occurrence.

Pirfenidone is one of the FDA-approved anti-fibrotic agents to treat idiopathic pulmonary fibrosis (IPF). Compared with methylprednisolone alone, pirfenidone and methylprednisolone combination therapy improves PF in hospitalized patients diagnosed with severe COVID-19 pneumonia (Acat et al., 2021). However, pirfenidone cannot prevent or reverse the progression of PF, which also limits its use in COVID-19/PF co-occurrence (Lancaster et al., 2019; Noble et al., 2011). There is no reported effective treatment for COVID-19/PF co-occurrence so far, thus the discovery of effective drugs against COVID-19/PF co-occurrence will contribute to improving patient prognosis and reducing social burdens. Surprisingly, it is confirmed that natural products have the effect of suppressing viral replication and transcription, and can inhibit cytokine storm and improve immunodeficiency (An et al., 2021). Natural product is also increasingly recognized as an alternative source for inhibiting fibrosis (Bahri et al., 2017). Natural products can reduce fibrosis by inhibiting inflammation, myofibroblast activation, ECM accumulation and epithelial-mesenchymal transition (EMT) (Chen et al., 2018). Natural products are a treasure trove for discovering new therapeutic drugs for COVID-19/PF co-occurrence. A natural product with dual antiviral and antifibrotic effects may have the great potential to become a therapeutic agent for COVID-19 and PF comorbidities.

**FIGURE 1:**
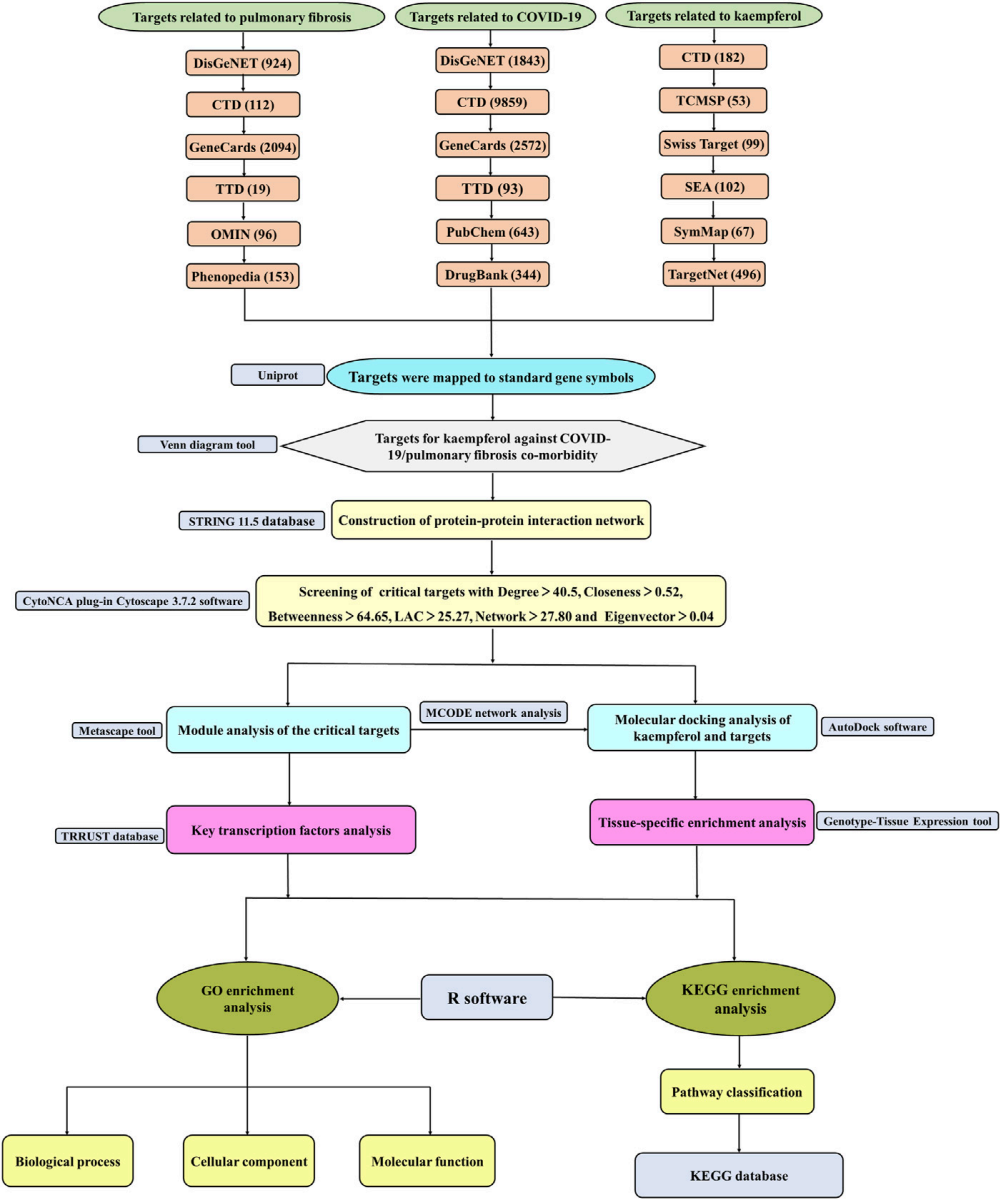
The flow diagram of a pragmatic strategy for identifying the pharmacological mechanism of kaempferol against COVID-19/pulmonary fibrosis co-occurrence based on system pharmacology and bioinformatics analysis.

Kaempferol, a natural flavonoid that widely exists in many fruits, vegetables and herbal medicine, is known as an antimicrobial, anti-inflammatory and antioxidant compound (Devi et al., 2015; Imran et al., 2019; Ren et al., 2019). Main protease (Mpro), a potential drug target for treating COVID-19, is found to be potentially inhibited by kaempferol (Khaerunnisa et al., 2020; Mahmud et al., 2021). Moreover, it is reported that kaempferol may directly target SARS-CoV-2 main protease (3CL pro) to perform anti-COVID effect (Shaldam et al., 2021; Zhang et al., 2021). Simultaneously, kaempferol inhibits the progression of silica-induced PF and attenuates fibrotic airway remodeling *via* modulating protease-activated receptor-1 activation (Gong et al., 2014; Liu et al., 2019). The above researches suggest that kaempferol has dual effects against COVID-19/PF co-occurrence, but the molecular mechanisms have not been investigated. Therefore, drug-target, disease-target and critical targets among COVID-19, PF and kaempferol were captured. Protein-protein interaction (PPI), MCODE, transcription factors, tissue-specific enrichment, molecular docking, Kyoto Encyclopedia of Genes and Genomes (KEGG) pathways and Gene Ontology (GO) analyses were performed. The detailed strategy of exploring the targets and mechanisms of kaempferol against COVID-19/PF co-occurrence by bioinformatics and network pharmacology is shown in Figure 1.

## Materials and Methods

### Screening for Drug-Related Targets

The targets associated with kaempferol were retrieved from Comparative Toxicoomics Database (CTD, http://ctdbase.org/, accessed date: 3 September 2021) (Davis et al., 2021), Traditional Chinese Medicine Systems Pharmacology Database and Analysis Platform (TCMSP, https://tcmspe.com/, accessed date: 2 September 2021) (Ru et al., 2014), Swiss Target Prediction (http://swisstargetprediction.ch/, accessed date: 2 September 2021) (Daina et al., 2019), Similarity Ensemble Approach (SEA, https://sea.bkslab.org/, accessed date: 2 September 2021) (Keiser et al., 2007), SymMap (https://www.symmap.org/, accessed date: 2 September 2021) (Wu et al., 2019) and TargetNet (http://targetnet.scbdd.com/, accessed date: 2 September 2021) (Yao et al., 2016).

### Collection of Disease-Related Targets

Targets related to COVID-19 were obtained from DisGeNET (https://www.disgenet.org/home/, accessed date: 2 September 2021) (Pinero et al., 2017), CTD, GeneCards (https://www.genecards.org/, accessed date: 3 September 2021) (Rebhan et al., 1997), Therapeutic Target Database (TTD, http://db.idrblab.net/ttd/, accessed date: 3 September 2021) (Wang et al., 2020), PubChem (https://pubchem.ncbi.nlm.nih.gov/, accessed date: 2 September 2021) (Kim et al., 2021) and DrugBank database (https://www.drugbank.com/, accessed date: 4 September 2021) (Wishart et al., 2018).

Six databases were used to obtained PF-related targets including DisGeNET, CTD, GeneCards, TTD, Online Mendelian Inheritance in Man (OMIM, https://omim.org/, accessed date: 2 September 2021) (Amberger et al., 2015) and Phenopedia (https://phgkb.cdc.gov/PHGKB/startPagePhenoPedia.action, accessed date: 2 September 2021) (Yu et al., 2010). Targets were mapped to standard symbols by using Uniprot database (https://www.uniprot.org/, accessed date: 2 September 2021) (UniProt, 2015).

### Analysis of Overlapping Targets Between Drug and Diseases

The Venn package of R 3.6.2 software was used to draw the petal map. Venn diagram showing the intersection of the targets of kaempferol against COVID-19/PF co-morbidity was plotted by the Venn Diagram tool (http://bioinformatics.psb.ugent.be/webtools/Venn/) and Microsoft Excel.

### Protein-Protein Interaction Network Construction and Critical Targets Analysis

The shared targets between diseases and drug were put into the STRING 11.5 database (https://string-db.org/, accessed date: 6 September 2021) (Szklarczyk et al., 2021) to construct a PPI network. The organism was set to “*Homo sapiens*” and the minimum required interaction score was set to 0.4. Then the PPI network was visualized by Cytoscape 3.7.2 software (https://cytoscape.org/) (Otasek et al., 2019). The CytoNCA plug-in Cytoscape 3.7.2 software was applied to calculate topological parameters including degree, closeness, betweenness, LAC, network and eigenvector (Tang et al., 2015). Regarding the medians of topological parameters as the screening threshold, the overlapping targets above the threshold were identified as critical targets.

### Module Analysis of Critical Targets

Metascape (http://metascape.org/, accessed date: 7 September 2021) was used to perform module analysis of critical targets (Zhou et al., 2019). MCODE score (Bader and Hogue, 2003) was applied to cluster the most significant modules. Code score was calculated on the connection density of the adjacent area, and the target in MCODE module with greater degree value was considered to play a more important role in treating COVID-19/PF co-morbidity. Of note, the top five targets with the highest degree values in MCODE modules were selected to perform molecular docking analysis.

### Key Transcription Factors Analysis of Critical Targets

Transcriptional Regulatory Relationships Unraveled by Sentence-based Text mining (TRRUST, https://www.grnpedia.org/trrust/, accessed date: 7 September 2021) is a useful tool for predicting transcriptional regulatory network (Han et al., 2018). The TRRUST database provides abundant information of 8,444 transcription factors (TFs)-target network. Critical targets were input to TRRUST database with the species of “Human.” The top 10 TFs ranking based on *p* value from small to large were selected to construct the TFs-target network by using Cytoscape 3.7.2 software.

### Tissue-Specific Enrichment Analysis of Critical Targets

Genotype-Tissue Expression (GETx) (https://www.gtexportal.org/, accessed date: 7 September 2021) is an online tool to study genetic variation and expression of human tissues (Consortium, 2013). The top 50 targets ranking based on modules’ degree values from high to low were selected for tissue-specific enrichment analysis. The heat map showed the correlation between different samples and targets, and more the important tissues corresponding to the targets would show darker colors.

### Gene Ontology and Kyoto Encyclopedia of Genes and Genomes Enrichment Analyses of Critical Targets

GO enrichment analysis included biological process (BP), molecular function (MF) and cellular component (CC), as well as KEGG pathway enrichment analysis were conducted in R 3.6.2 software. “Org.hs.eg.db” (https://www.bioconductor.org/packages/org.Hs.eg.db, accessed date: 7 September 2021) was used to match the gene ID corresponding to critical targets. Then “cluster Profiler” package (Wu et al., 2021) was used to perform enrichment analysis with the criteria of *p*valueCutoff = 0.05 and *q*valueCutoff = 0.05. Based on adjusted *p* value in ascending order, the top 20 enrichment results were selected to display as a bubble chart by bioinformatics tool (http://www.bioinformatics.com.cn/). Furthermore, the KEGG pathways were classified based on KEGG databases and visualized by hiplot (https://hiplot.com.cn/).

### Molecular Docking Analysis of the Top Five Targets

Molecular docking is widely applied in drug detection and is often used to predict the relationship between targets and ligand. Molecular docking was carried out between kaempferol and the top five targets *via* AutoDock software (Vina 1.5.6, http://autodock.scripps.edu/) (Shen et al., 2021; Trott and Olson, 2010), which was often used to calculate the molecular interaction force between protein and ligand. The small-molecule two-dimensional structure format information of kaempferol was obtained from PubChem database (https://pubchem.ncbi.nlm.nih.gov/) and saved in the SDF format. The SDF molecular structure file of kaempferol was converted into a PDB file by Open Babel software. The three-dimensional structure of key target proteins was downloaded from the RCSB PDB database (https://www.rcsb.org/) (Rose et al., 2021). The Auto Dock Tools 1.5.6 software was used to convert the molecular structure document into PDBQT format and perform molecular docking. The PyMol 2.3.2 software was used to visualize the results with higher docking scores and calculate the corresponding RMSD values.

## Results

### Targets of Kaempferol Against COVID-19/PF Co-Occurrence

**FIGURE 2:**
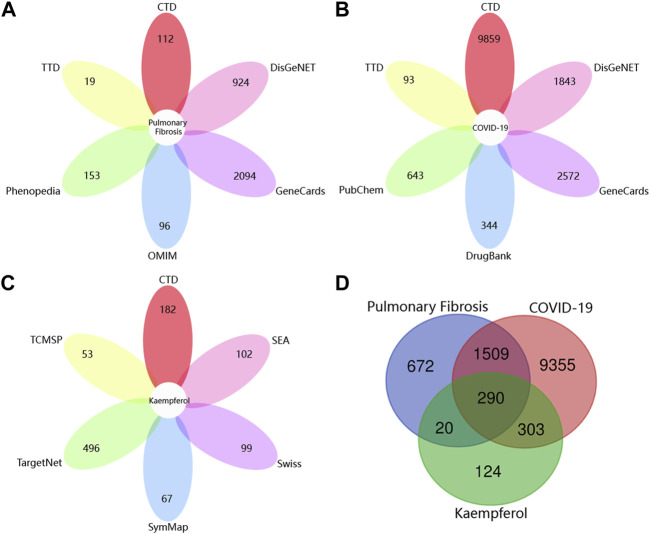
Collection of targets related to drug and diseases from various open-source databases. **(A)** The number of pulmonary fibrosis-related targets from six open-source databases. **(B)** The number of targets related to COVID-19 from six open-source databases. **(C)** The number of targets associated with kaempferol from six open-source databases. **(D)** Venn diagram depicting common targets between COVID-19, pulmonary fibrosis and kaempferol.

As shown in Figure 2A unique PF-related targets were obtained from DisGeNET (924), CTD (112), GeneCards (2,094), TTD (19), OMIM (96) and Phenopedia (153). As shown in Figure 2B, 11,457 unique targets of COVID-19 were retrieved from DisGeNET (1,843), CTD (9,859), GeneCards (2,572), TTD (93), PubChem (643) and DrugBank (344). As shown in Figure 2C, 737 unique targets related to kaempferol were identified from CTD (182), TCMSP (53), Swiss Target Prediction (99), SEA (102), SymMap (67) and TargetNet (496). Finally, 290 targets of kaempferol against COVID-19/PF co-occurrence were obtained (Figure 2D).

### Protein-Protein Interaction Network Construction and Critical Targets Acquisition

**FIGURE 3:**
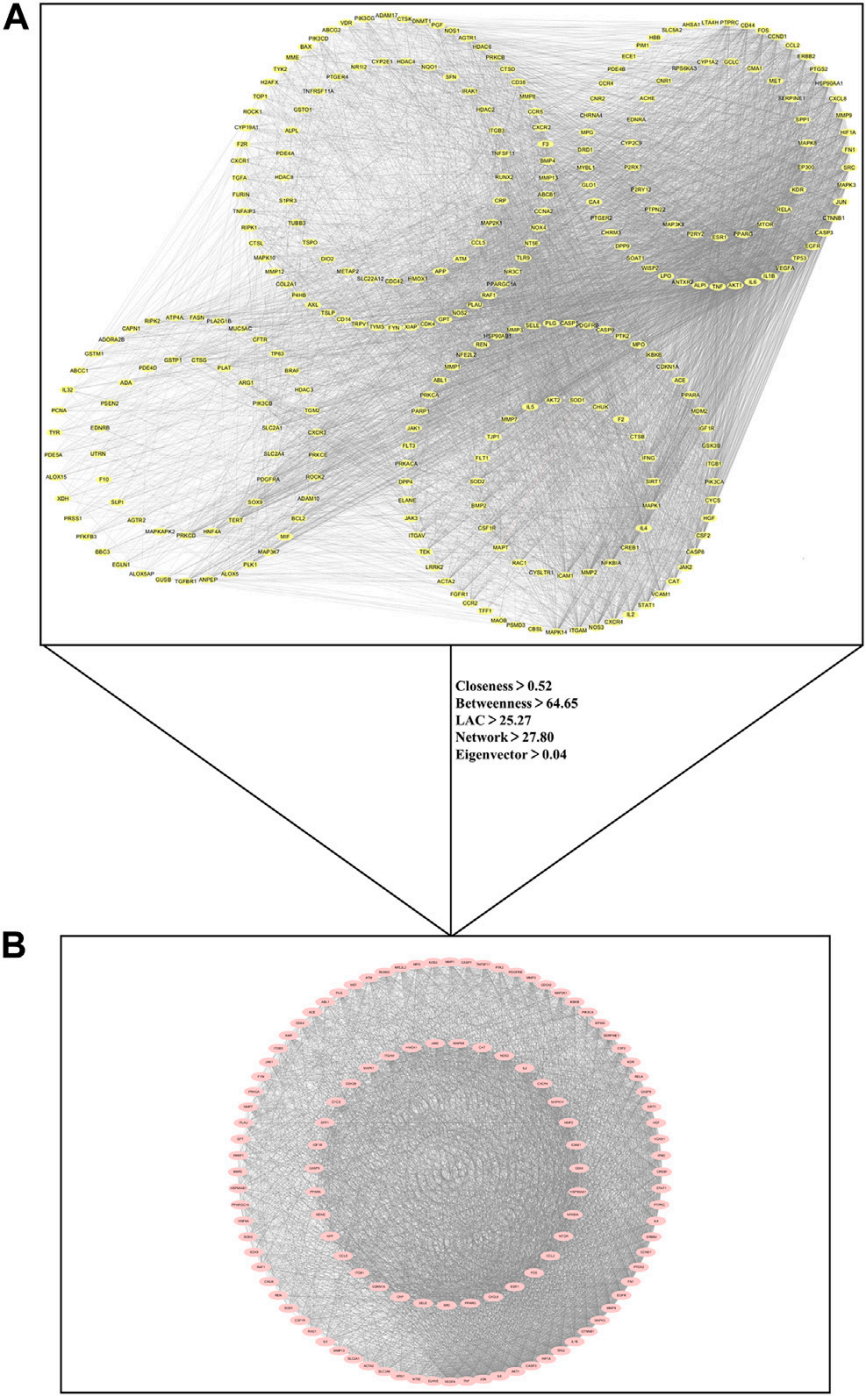
Protein-protein interaction (PPI) network for critical targets of kaempferol against COVID-19/pulmonary fibrosis co-occurrence. Nodes represent targets and edges represent protein-protein interactions. **(A)** PPI network of 290 common targets between COVID-19, pulmonary fibrosis and kaempferol. **(B)** PPI network of 115 critical targets for kaempferol against COVID-19/pulmonary fibrosis co-occurrence.

The nodes represented shared targets and the edges indicated protein-protein interactions between shared targets in PPI network. PPI network of 290 common targets shown in Figure 3A contained 290 nodes and 7,431 edges. Through the topological identification and calculation of PPI network, the medians of the topological parameter were degree = 40.5, closeness = 0.52, betweenness = 64.65, LAC = 25.27, network = 27.80 and eigenvector = 0.04. Then 115 critical targets with the topological parameters greater than the medians of above six topological factors were screened out to construct PPI network of critical targets. There were 115 nodes and 3,639 edges in the PPI network of critical targets as shown in Figure 3B.

### Investigation of Important Modules

**FIGURE 4:**
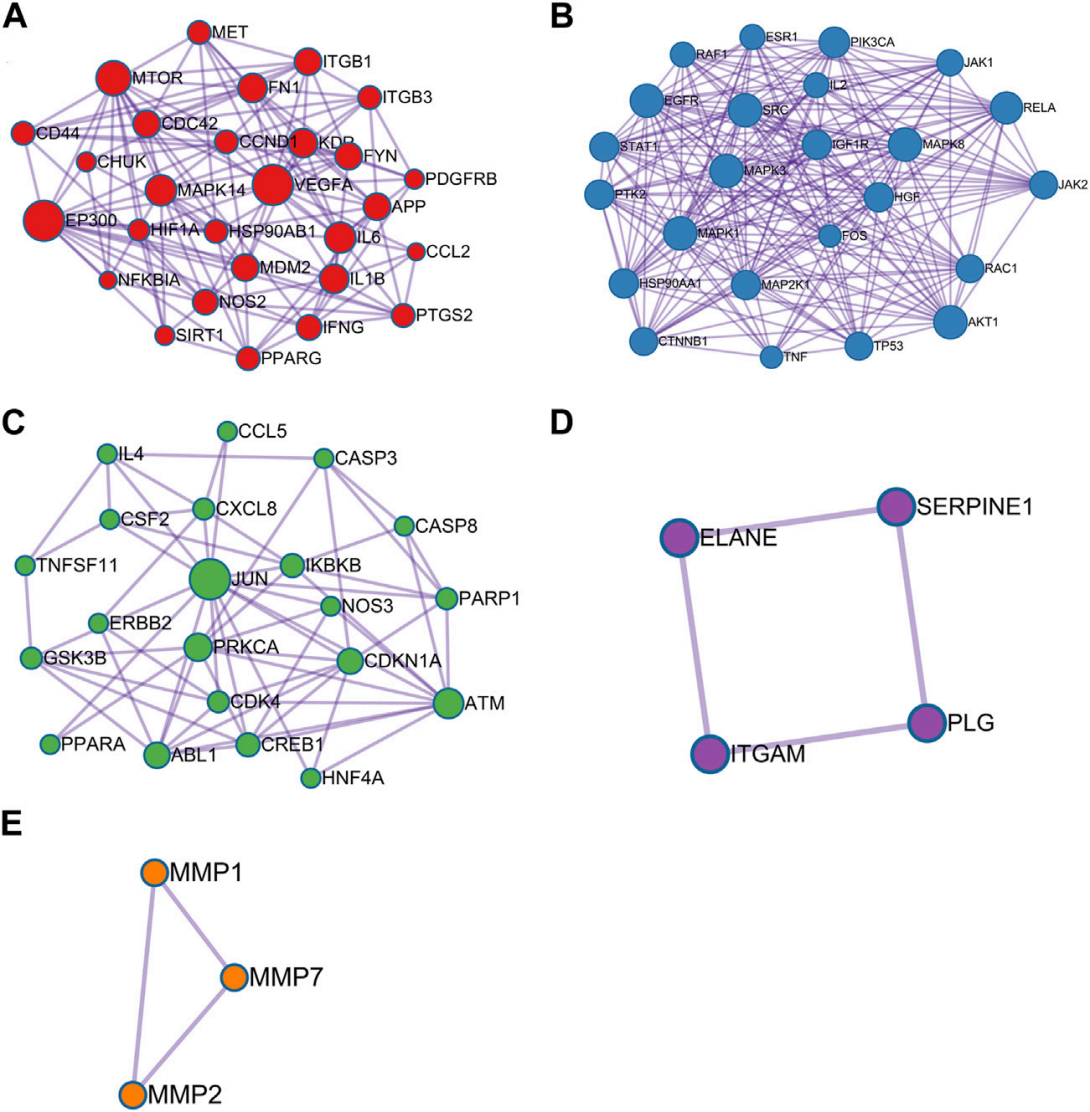
Module analysis of 115 critical genes is performed by the Metascape tool. Each module demonstrats different biological process functions. **(A)** Module 1; **(B)** Module 2; **(C)** Module 3; **(D)** Module 4; **(E)** Module 5.

Module analysis was carried out by using Metascape tool and five functional clusters were shown in Figures 4A–E. Module 1 included 28 nodes and 132 edges with MCODE score = 4.714. Module 2 contained 24 nodes and 205 edges with MCODE score = 8.541. Module 3 included 21 nodes and 62 edges with MCODE score = 2.952. Module 4 comprised of 4 nodes and 4 edges with MCODE score = 1.000. Module 5 included 3 nodes and 3 edges with MCODE score = 1.000. The top five targets with the highest degree scores were epidermal growth factor receptor (EGFR, degree = 23), proto-oncogene tyrosine-protein kinase SRC (SRC, degree = 21), mitogen-activated protein kinase 3 (MAPK3, degree = 21), mitogen-activated protein kinase 1 (MAPK1, degree = 21), mitogen-activated protein kinase 8 (MAPK8, degree = 20), RAC-alpha serine/thre onine-protein kinase (AKT1, degree = 20), transcription factor p65 (RELA, degree = 19) and phosphatidylinositol 4,5-bisphosphate 3-kinase catalytic subunit alpha isoform (PIK3CA, degree = 18).

### Key Transcription Factors Acquisition

**FIGURE 5:**
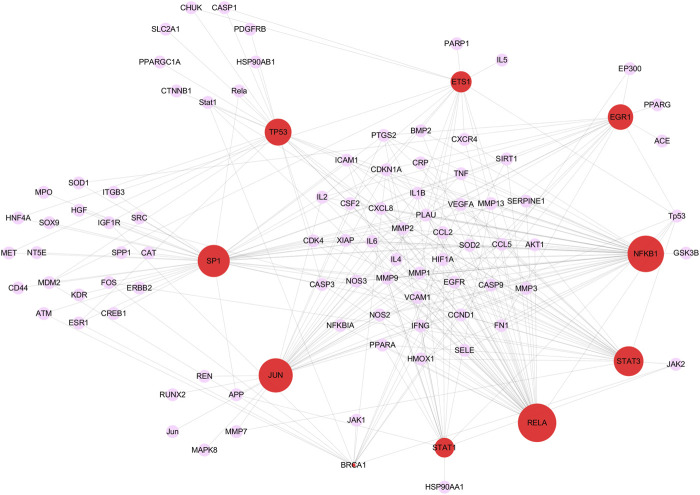
The top 10 key transcription factors (TFs) of 115 critical targets. The red nodes represent TFs and the purple nodes represent corresponding targets. The edges represent the connection between TFs and targets. The sizes of red nodes present negative correlation with *p* values and a node with larger shape represents the more important role in treating COVID-19/pulmonary fibrosis co-occurrence.

115 critical targets were input to the TRRUST database and 203 TFs were obtained. TFs-target network contained 97 nodes including 10 TFs, 87 targets and 278 edges (Figure 5). Red nodes represented TFs and purple nodes represented corresponding targets, and the edge indicated the relevance between TFs and corresponding targets. The size of the red node was negatively correlated with *p* value, the larger the size of the red node was, the more important it is in the TFs-target network. Especially, there were four critical targets that were also predicted as TFs, including signal transducerand activator of transcription 1 (STAT1), tumor protein P53 (TP53), JUN proto-oncogene, AP-1 transcription factor subunit (JUN) and RELA. The detailed information of the top 10 TFs were listed in Table 1.

**TABLE 1:** Key transcription factors associated with critical targets.

Key transcription factors	Description	*p* value
RELA	V-rel *reticuloendotheliosis* viral oncogene homolog A (avian)	1.33E-49
NFKB1	Nuclear factor of kappa light polypeptide gene enhancer in B cells 1	3.34E-46
JUN	Jun proto-oncogene	2.28E-41
SP1	Sp1 transcription factor	2.52E-39
STAT3	Signal transducer and activator of transcription 3 (acute-phase response factor)	2.7E-33
TP53	Tumor protein p53	1.03E-26
EGR1	Early growth response 1	5.56E-23
ETS1	V-ets *erythroblastosis virus* E26 oncogene homolog 1 (avian)	3.68E-22
STAT1	Signal transducer and activator of transcription 1, 91 kDa	1.15E-21
BRCA1	Breast cancer 1, early onset	4.04E-21

### Critical Targets Were Mostly Enriched in Lung Tissue

**FIGURE 6:**
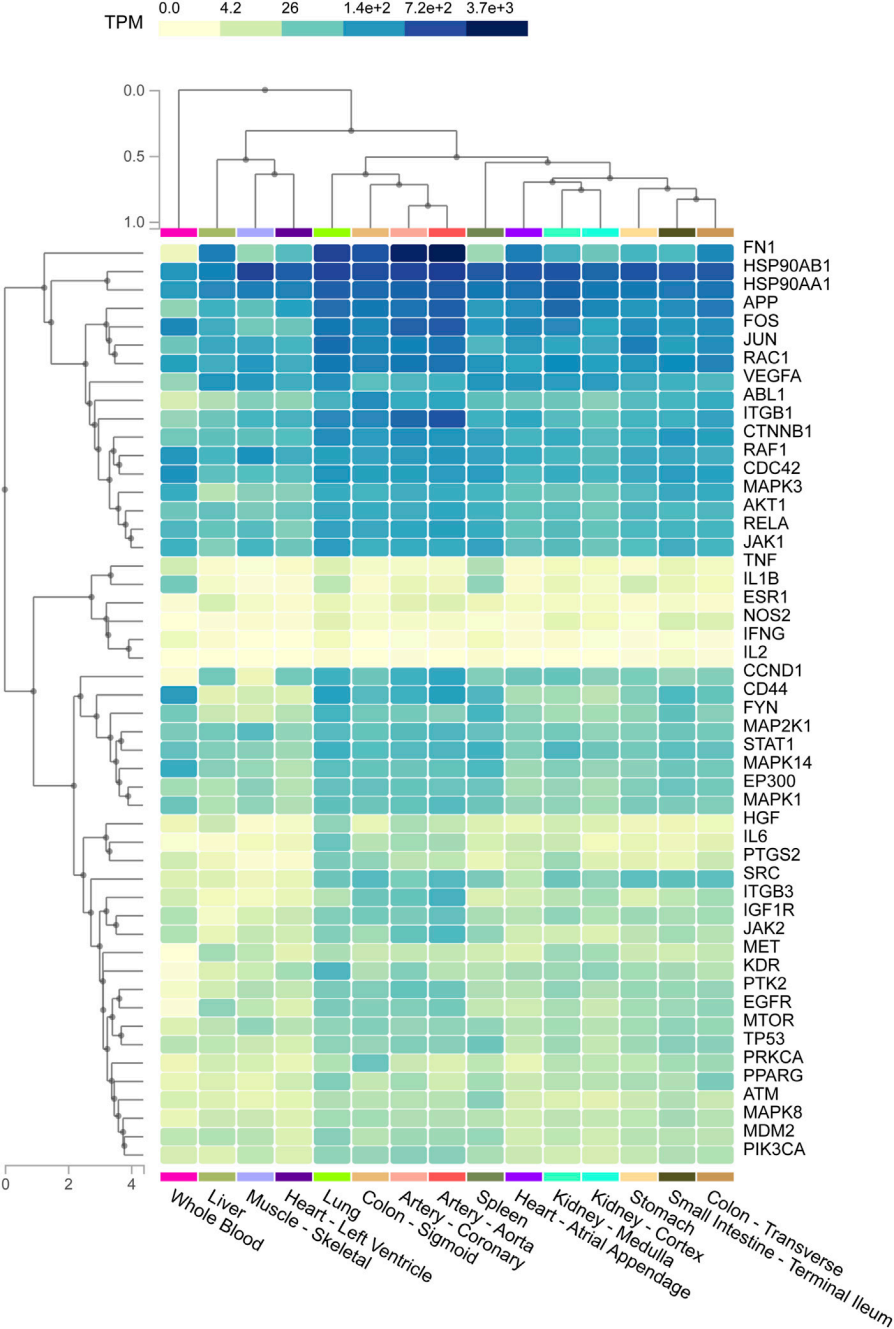
The heat map shows the relationship between different tissue samples and critical targets. Column represents critical targets and row represents enriched tissues. The shades of colors represent the levels of enrichment of critical targets in tissues, and the darker the color indicates the more significant enrichment of targets in corresponding tissues.

Tissues were represented on the abscissa and targets were indicated on the ordinate (Figure 6). The data was presented as a heat map and the color indicated the level of enrichment. The darker the color was, the higher the expression level of critical target in corresponding tissue was. The result indicated that most critical targets were highly expressed in lung tissue, especially fibronectin 1 (FN1), heat shock protein 90 alpha family class B member 1 (HSP90AB1), fos proto-oncogene, AP-1 transcription factor subunit (FOS), JUN, RAC family small GTPase 1 (RAC1), vascular endothelial growth factor A (VEGFA), ABL proto-oncogene 1, non-receptor tyrosine kinase (ABL1), RELA, heat shock protein 90 alpha family class A member 1 (HSP90AA1) and so on.

### Gene Ontology Enrichment Analysis

**FIGURE 7:**
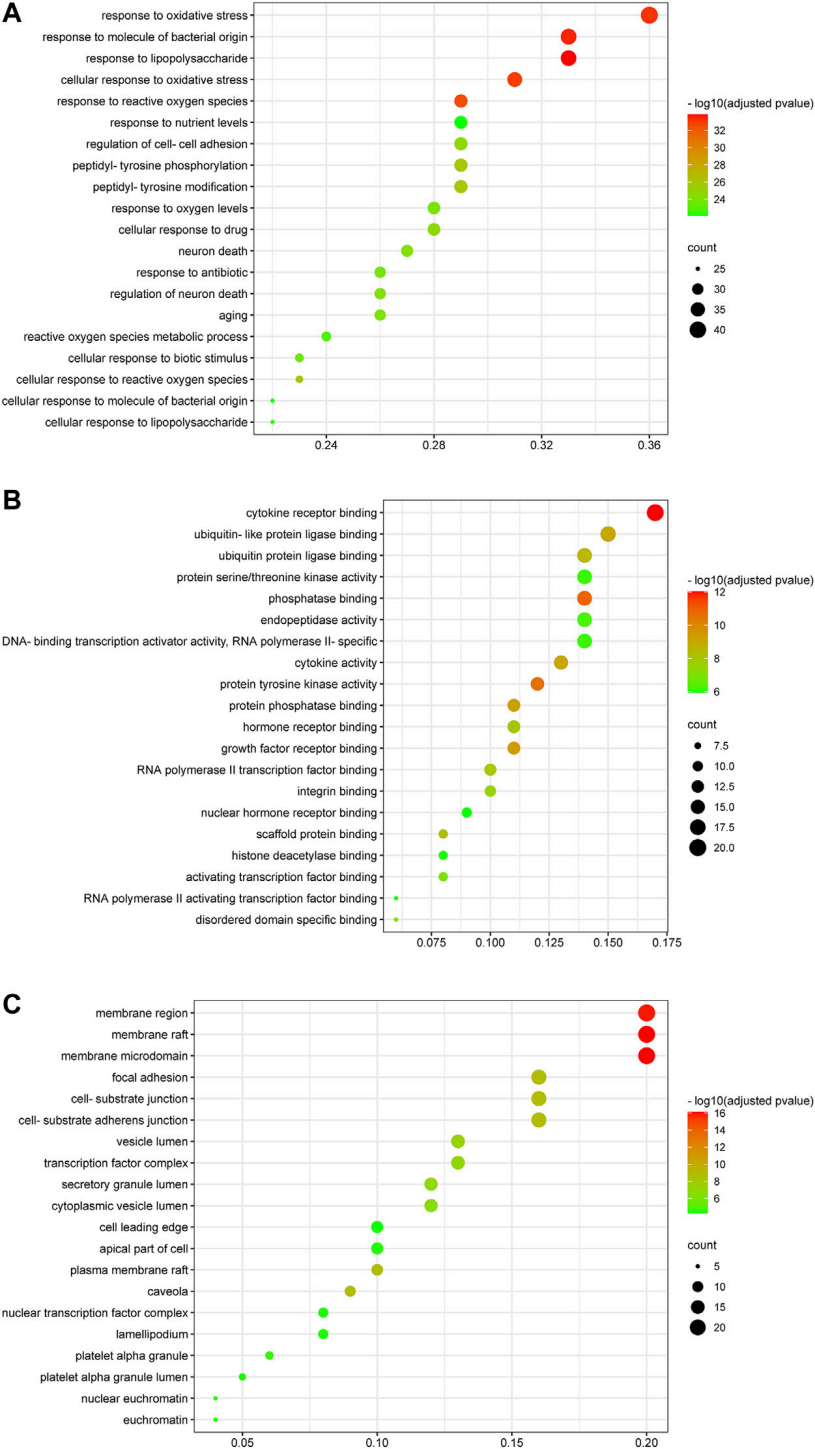
Gene ontology enrichment analysis of critical targets. The size of the node represents the number of genes involved in the GO term, and the color from green to red indicates the −log10 (adjusted *p* value) from small to large. **(A)** Biological process enrichment results of critical targets. **(B)** Molecular function enrichment results of critical targets. **(C)** Cellular components enrichment results of critical targets.

2,958 GO terms were obtained, of which 2,705 belonged to GO-BP, 94 to GO-CC and 159 to GO-MF. The top 20 GO terms were respectively shown in Figures 7A–C. As for GO-BP, critical targets were mainly enriched in response to lipopolysaccharide, response to molecule of bacterial origin, response to oxidative stress, cellular response to biotic stimulus, response to antibiotic, regulation of cell-cell adhesion and so on. As for GO-MF, critical targets were mainly enriched in cytokine receptor binding, phosphatase binding, protein tyrosine kinase activity, growth factor receptor binding, protein phosphatase binding and so on. As for GO-CC, critical targets were mainly enriched in membrane raft, membrane microdomain, membrane region, focal adhesion, cell-substrate adherens junction and so on.

### Kyoto Encyclopedia of Genes and Genomes Enrichment Analysis

**FIGURE 8:**
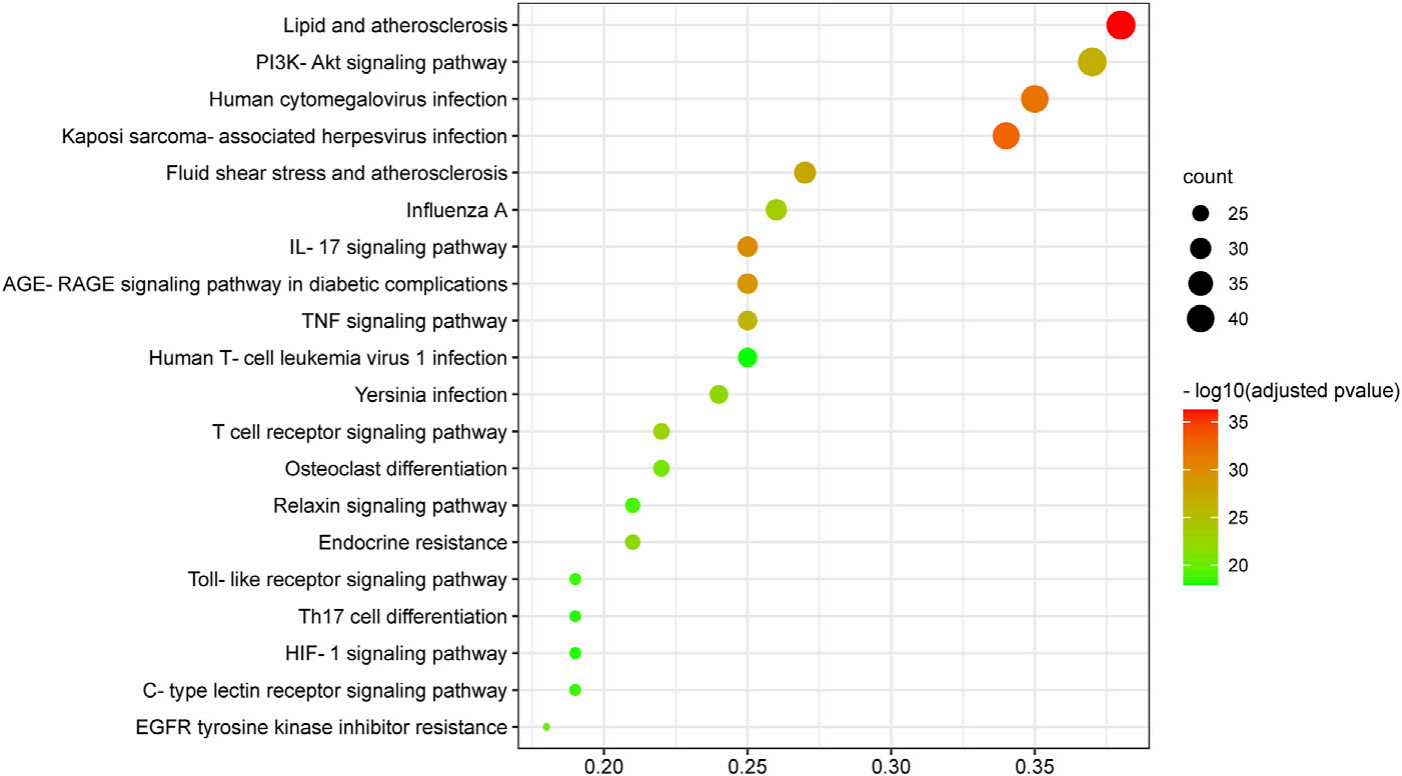
Kyoto Encyclopedia of Genes and Genomes enrichment analysis of critical targets. The size of the node represents the number of genes involved in the enrichment pathway, and the color from green to red indicates the −log10 (adjusted *p* value) from small to large.

**FIGURE 9:**
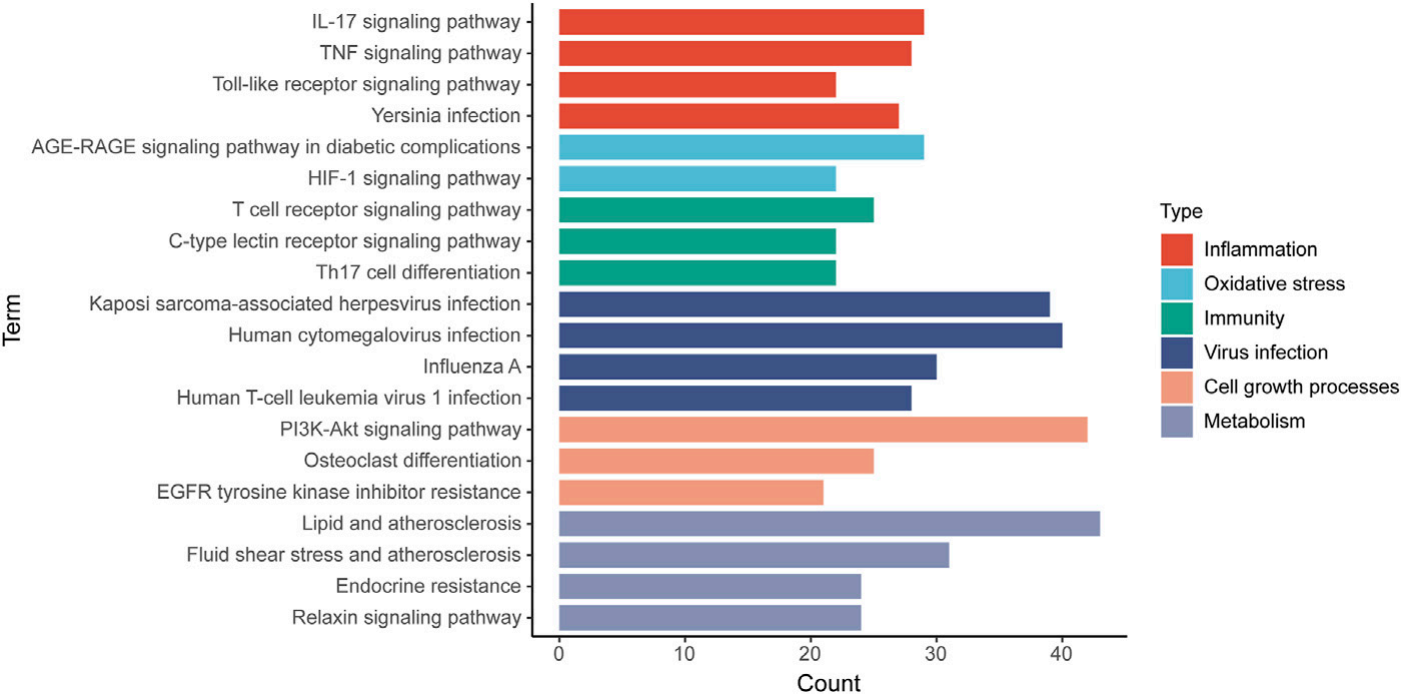
The classification of KEGG pathways. The results of KEGG pathway enrichment analysis are classified into five types and a color represents a type. Column represents KEGG pathway terms and row represents the number of targets enriched on the pathways.

174 KEGG terms were acquired and the top 20 KEGG terms were shown in Figure 8. Critical targets were mainly enriched in the EGFR tyrosine kinase inhibitor resistance, interleukin 17 (IL-17) signaling pathway, tumor necrosis factor (TNF) signaling pathway, Toll-like receptor signaling pathway, Yersinia infection, advanced glycation end product-receptor for advanced glycation end product (AGE-RAGE) signaling pathway in diabetic complications, hypoxia inducible factor 1 (HIF-1) signaling pathway, T cell receptor signaling pathway, C-type lectin receptor signaling pathway, Th17 cell differentiation, phosphoinositide 3-kinase/AKT serine/threonine kinase (PI3K/Akt) signaling pathway and so on. The results of KEGG pathway enrichment analysis were classified into five types, containing inflammation, oxidative stress, immunity, virus infection, cell growth processes and metabolism (Figure 9).

### Kaempferol Had Good Binding Activities With Critical Targets

**FIGURE 10:**
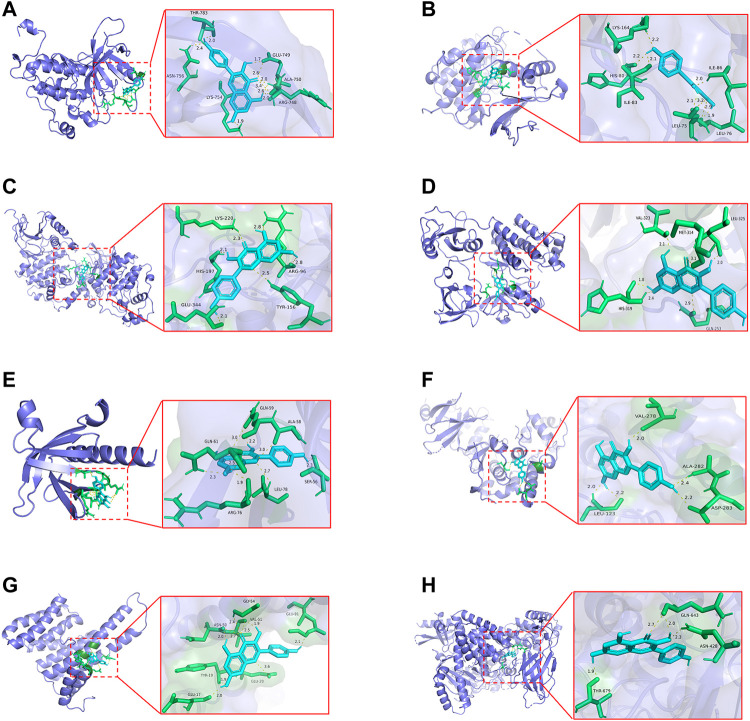
The docking models of kaempferol with the identified the top eight critical targets. **(A)** Docking results of kaempferol and EGFR. **(B)** Docking results of kaempferol and MAPK1. **(C)** Docking results of kaempferol and MAPK3. **(D)** Docking results of kaempferol and SRC. **(E)** Docking results of kaempferol and AKT1. **(F)**Docking results of kaempferol and MAPK8. **(G)**Docking results of kaempferol and RELA. **(H)**Docking results of kaempferol and PIK3CA.

To investigate whether kaempferol directly binds to EGFR, MAPK1, MAPK3, SRC, AKT1, MAPK8, RELA and PIK3CA (the top five targets with the highest degree values), molecular docking analysis was performed by Auto Dock Tools software. A binding energy less than 0 indicates spontaneous binding of ligand and receptor. The lower binding energy indicates a better binding effect. It is generally believed that binding energy < −5 kcal mol^−1^ indicates a good binding activity. Moreover, the stability of the simulated molecular docking systems was investigated by the root-mean-square deviation (RMSD), and it means the system is stable when RMSD is lower than 2 Å. The molecular docking results showed that the binding energies of kaempferol and the eight critical targets ranged from −6.23 to −8.15 kcal mol^−1^ (Table 2). All the simulated molecular docking reached the RMSD value range required for stability. The better docking result was selected for molecular docking visualization by using PyMol 2.3.2 software. The results showed that 2-5 hydrogen bonds could be formed between kaempferol and the eight critical targets (Figure 10). Molecular docking results proved that kaempferol had good binding activities with the eight critical targets.

**TABLE 2:** Molecular docking results of kaempferol with top eight critical targets.

Number	Target protein	PDB ID	RMSD	Binding energy (kcal/mol)
1	EGFR	5HG8	0.263	−7.530
2	MAPK1	6SLG	0.000	−6.170
3	MAPK3	4QTB	0.024	−6.750
4	SRC	1FMK	0.838	−7.720
5	AKT1	1UNQ	0.000	−6.550
6	MAPK8	2XRW	0.010	−6.420
7	RELA	6NV2	0.002	−6.230
8	PIK3CA	6PYS	0.048	−8.150

## Discussion

The prevention and treatment of COVID-19 related complications are public concerns. COVID-19/PF co-occurrence is a common and threatening condition, and early intervention is important for improving prognosis of pulmonary complications caused by SARS-CoV-2 infection (Pan et al., 2020). Traditional natural products have the effect of inhibiting viral replication and transcription, reducing cytokine storm and ameliorating immunodeficiency (An, et al., 2021). Further, growing evidence shows that natural products are alternative sources for improving fibrosis (Bahri, et al., 2017). Therefore, it reveals that natural product is a treasure trove for discovering new therapeutic drugs. It has been confirmed that kaempferol alleviates H9N2 influenza virus-induced inflammation and acute lung injury (Zhang et al., 2017). Kaempferol can also inhibit the virus replication of the pseudorabies virus in mice (Li et al., 2021). Moreover, kaempferol is proved to inhibit the activity of the Japanese encephalitis virus in BHK-21 cells (Care et al., 2020). Except for the antiviral effect, the anti-PF effect of kaempferol is also verified by a silica-induced PF mice model (Liu, et al., 2019). The above evidences indicate that kaempferol with dual antiviral and anti-PF effects may be the promising medicine for treating COVID-19/PF co-occurrence. Thus, this study analyzed potential targets and mechanisms of kaempferol against COVID-19/PF co-occurrence by integrating bioinformatics and system pharmacological tools.

First, 290 common targets between kaempferol, COVID-19 and PF were obtained, and then 115 critical targets with greater topological parameters in the PPI network were screened out. The top five targets from the 115 critical targets were identified, including EGFR (degree = 23), SRC (degree = 21), MAPK3 (degree = 21), MAPK1 (degree = 21), MAPK8 (degree = 20), AKT1 (degree = 20), RELA (degree = 19) and PIK3CA (degree = 18). Computer modelling approaches show that kaempferol has a high binding affinity to 3CLpro (Shaldam, et al., 2021; Zhang, et al., 2021). *In vitro* experiment confirms that kaempferol has strong inhibitory effects on 3CLpro (Khan et al., 2021). Of note, except for the direct effect on virus-produced proteins, downstream molecules or signaling pathways during the pathologic process are also potential mechanisms for kaempferol against COVID-19/PF co-occurrence. Surprisely, molecular docking analysis found that kaempferol showed promising binding activities with the top five targets (EGFR, SRC, MAPK3, MAPK1, MAPK8, AKT1, RELA and PIK3CA). EGFR inhibitors are proved to have antiviral and antifibrotic effects based on the Viral Fibrotic score, indicating that EGFR may be a critical regulator of COVID-19/PF co-occurrence (Vagapova et al., 2021). SRC is involved in the pathogenesis of PF by regulating EMT, myofibroblast differentiation and inflammation.

Xu et al. (2020), and a recent study reports that targeting SRC reduces titers of SARS-CoV-2 (Meyer et al., 2021). AKT shows an increased trend in various fibrotic diseases (Lu et al., 2010; Huang et al., 2011), and it also increases in fibroblasts of bleomycin-induced IPF *in vivo* and *in vitro* (Vittal et al., 2005; Xia et al., 2008; Le Cras et al., 2010). Moreover, deficiency of AKT1 significantly inhibits viral RNA expression (Esfandiarei et al., 2004), and PI3K/AKT kinase inhibitors are found to suppress the replication of middle east respiratory syndrome (MERS) (Kindrachuk et al., 2015). The first identified member of the MAPK pathway is extracellular signal-regulated kinase (ERK)1/2, which overexpresses in IPF (Antoniou et al., 2010). A study confirms that inhibition of ERK1/2 attenuates bleomycin-mediated PF by inhibiting EMT (Zou et al., 2020). In addition, MAPK is also involved in regulating virus replication, immune response and apoptosis of virus-infected cells (Bian et al., 2011; Gaur et al., 2011). It is worth noting that p38 MAPK inhibitor effectively prevents the phosphorylation of heat shock protein 27, cathelicidin antimicrobial peptide response element-binding protein and eukaryotic initiation factor 4E in SARS-CoV infected cells (Mizutani et al., 2004). RELA regulates the interferon IFN response during SARS-CoV-2 infection (Yin et al., 2021), and inhibition of RELA contributes to improving PF (Hou et al., 2018). PIK3CA belongs to the lipid kinase family and is responsible for coordinating functions such as proliferation, vesicle trafficking, and protein synthesis in various cells (Maheshwari et al., 2017). The above results reveal that targeting the critical targets especially the top five targets may be the potential therapeutic approach for kaempferol against COVID-19/PF co-occurrence.

Abnormal TFs activation and subsequent abnormal pathogenic genes expression play important roles in disease progression. The top 10 TFs were identified from 115 critical targets, and RELA was the most significant TF with the smallest *p* value among the top 10 TFs. The activation of RELA, a subtype of nuclear factor kappa-B (NF-*κ*B), enhances the expression of TGF-β1 (Rameshwar et al., 2000). TGF-β1 is a key pro-fibrotic factor that has been proved to promote the transition of fibroblast to myofibroblast in PF (Andersson-Sjoland et al., 2008; Goodwin and Jenkins, 2009). ACT001 (NF-*ĸ*B inhibitor) attenuates PF through decreasing the transition of fibroblast to myofibroblast, inhibiting IL-6 production and fibronectin deposition (Jaffaret al., 2021). Increased inflammatory cytokines and chemokines levels result in spontaneous haemorrhage, thrombocytopenia and systemic inflammation, which are the main manifestations of the fatal cytokine syndrome in advanced COVID-19 patients (Song et al., 2020; Xu et al., 2020). The activation of NF-*ĸ*B enhances the expression of inflammatory cytokines and chemokines, including IL-1, IL-6, IL-8 and TNF-α (Liao et al., 2005; Wang et al., 2007). Selective bruton tyrosine kinase inhibitor inhibits NF-*ĸ*B at the RELA phosphorylation stage, which leads to the reduction of C-reactive protein and IL-6 and an improvement of oxygen saturation (Roschewski et al., 2020). Further, to explore the association between tissues and critical targets, tissue-specific enrichment analysis was performed. The results showed that FN1, HSP90AB1, HSP90AA1 and so on were significantly enriched in the lung tissues. One of the characteristics of PF is excessive deposition of ECM proteins such as fibronectin (Liu et al., 2017). Elevated fibronectin deposition has been found in the lung tissues of PF patients (Liu et al., 2019), and it has been suggested that SARS-CoV-2 infection may promote the fibronectin expression in alveolar epithelial cells (Xu et al., 2020). HSP90 plays an important role in the folding, maturation and stabilization of proteins, and is therefore required for replication of multiple DNA and RNA viruses (Nagy et al., 2011). HSP90 inhibitor could inhibit virus replication, thus inhibition of HSP90 may be an effective strategy against SARS-CoV-2 infection (Li et al., 2020). In addition, increasing evidence shows that HSP90 is closely related to fibrogenesis (Bellaye et al., 2014), and overexpression of HSP90 emerges as a hallmark pathological step indicating the fibrogenesis progress (Sontake et al., 2017; Bellaye et al., 2018). Immunohistochemistry study reveals that HSP90α and HSP90β are overexpressed in the lungs of IPF patients (Sibinska et al., 2017). HSP90α participates in the PF progress through promoting the phosphorylation of AKT in P38 and ERK signaling pathways (Dong et al., 2017). The above descriptions indicate that targeting critical targets and TFs to regulate downstream genes may contribute to improving the condition of COVID-19/PF co-occurrence.

The biological process and molecular mechanisms of critical targets were further analyzed by GO and KEGG enrichment analyses. Critical targets were found to be strongly associated with regulation of virus infection, oxidative stress, inflammation, immune response and metabolic process. One of the characteristics of oxidative stress is the excessive production of reactive oxygen species (ROS) that damage lung tissues over time (Otoupalova et al., 2020). In response to lung tissues damage, lung fibroblasts proliferate and migrate to the damaged area to differentiate into myofibroblasts, causing increased fibronectin, type I and III collagen (Thannickal et al., 2004). Furthermore, oxidative stress participates in the pathogenesis of COVID-19, and SARS-CoV-2 infection induces oxidative stress through increasing the production of ROS and inhibiting antioxidant capacity mediated by the nuclear factor erythroid 2-related factor 2 in the host (Olagnier et al., 2020). Unfortunately, raised oxidative stress will induce inflammatory cascades that ultimately lead to in apoptosis, lung injury and dysregulated of immune responses (Delgado-Roche and Mesta, 2020). The GO result suggests that the effect of kaempferol against COVID-19/PF co-occurrence may be closely associated with the regulation of biological process of oxidative stress, inflammation, immune response and metabolic process.

Furthermore, it was pleasant to find that critical targets were mainly involved in oxidative stress, inflammation, cell growth process, metabolism, immunity and virus infection-related pathways. Among the KEGG pathways, IL-17 signaling pathway, TNF signaling pathway, Toll-like receptor signaling pathway, HIF-1 signaling pathway, EGFR tyrosine kinase inhibitor resistance and PI3K/Akt signaling pathway showed significant significance. IL-17 is found to be highly expressed in patients with COVID-19 and PF comorbidities (Nuovo et al., 2012; Jahaj et al., 2021). Circulating IL-17 is overexpressed in severe COVID-19 patients compared to severe non-COVID-19 patients (Jahaj, et al., 2021). IL-17 signaling pathway is closely related to T helper (Th)17 cell differentiation and exacerbates cytokine storm during SARS-CoV-2 infection (Wu and Yang, 2020). High levels of IL-17 are also found in the lung tissues of IPF patients, which demonstrates that IL-17 signaling pathway is related to IPF progress (Nuovo, et al., 2012). TLRs are pattern recognition receptors involved in the PF process by regulating inflammation and injury repair (Kim et al., 2011). Moreover, activation of Toll-like receptor signaling pathway promotes the overexpression of pro-inflammatory factors (Conti et al., 2020). And interaction between TLRs and viral particles is one of the reasons that causes death of COVID-19 patients (Patra et al., 2021). HIF-1, an important transcriptional factor in response to hypoxia, plays an important role in mammalian oxygen homeostasis and is involved in PF progress (Epstein et al., 2001; Xiong and Liu, 2017). Selective silence of HIF-1α in alveolar epithelial cells can inhibit the progression of bleomycin-induced PF (Weng et al., 2014). Dysregulation of HIF exacerbates edema and inflammation in the lung tissues of patients with ALI, which is associated with glycolysis and mitochondrial respiration (Eckle et al., 2013). Other study also shows that the viral ORF3a protein increases the expression of HIF-1α, which in turn aggravates SARS-CoV-2 infection and inflammatory response (Tian et al., 2021). Besides, EGFR has dual pro-fibrotic and anti-fibrotic effect, and cancer patients treated with EGFR tyrosine kinase inhibitors-monoclonal antibody present an elevated incidence of interstitial lung disease (Osawa et al., 2015). However, a study suggests that gefitinib can inhibit the progression of mice models of bleomycin-induced PF (Ishii et al., 2006). Spontaneous PF is observed in transgenic mice with high expression of EGFR ligands (Korfhagen et al., 1994; Perugorria et al., 2008), and EGFR ligands silencing contribute to improving PF (Madtes et al., 1999). In a word, these studies show that abnormal EGFR expression promotes the development of PF. Moreover, EGFR inhibits IFN-I production (Lupberger et al., 2013) and significantly increases during ALI (Finigan et al., 2012), indicating that EGFR is a potential targeted pathway for treating COVID-19. ALI caused by cytokine storm is the characteristic of COVID-19, and only the combination of TNF-α (an important subtype of TNF signaling pathway) and IFN-γ can induce inflammatory cell death during SARS-CoV-2 infection (Karki et al., 2021). In addition, TNF-α significantly increases in mice models of bleomycin-induced PF (Hou, et al., 2018). PI3K/AKT kinase inhibitors are confirmed to inhibit the replication of MERS (Kindrachuk, et al., 2015), and the inhibition of PI3K/AKT signaling pathway contributes to alleviating PF (Fang et al., 2020). The above researches illustrate that COVID-19 and PF share the common targeting pathways, and IL-17, TNF, HIF-1, EGFR, PI3K/AKT and Toll-like receptor signaling pathways were the critical mechanisms of kaempferol against COVID-19/PF co-occurrence.

## Conclusion

This study is the first to elucidate the effect of kaempferol against COVID-19/PF co-occurrence by bioinformatics and systems pharmacology tools. The underlying mechanisms of kaempferol against COVID-19/PF co-occurrence may be related to bind to EGFR, SRC, MAPK3, MAPK1, MAPK8, AKT1, RELA and PIK3CA. Kaempferol might regulate inflammation, oxidative stress, immunity, virus infection, cell growth process and metabolism through targeting EGFR, IL-17, TNF, HIF-1, PI3K/AKT and Toll-like receptor signaling pathways to perform anti-COVID-19/PF co-occurrence effect. These findings suggest the possibility that kaempferol is a candidate compound to treat COVID-19/PF co-occurrence, but clinical, *in vivo* and *in vitro* experiments are needed to carry out to verify the predicted effect of kaempferol on COVID-19/PF co-occurrence in the future. This study contributes to providing effective strategy for exploring therapeutic approach for COVID-19/PF co-occurrence.

## Data Availability

The original contributions presented in the study are included in the article/Supplementary Material, further inquiries can be directed to the corresponding authors.
